# Identification and Characterization of Novel Small-Molecule Inhibitors against Hepatitis Delta Virus Replication by Using Docking Strategies

**DOI:** 10.5812/kowsar.1735143X.737

**Published:** 2011-10-01

**Authors:** Sarita Singh, Sunil Kumar Gupta, Anuradha Nischal, Sanjay Khattri, Rajendra Nath, Kamlesh Kumar Pant, Prahlad Kishore Seth

**Affiliations:** 1 Bioinformatics Center, Biotech Park, Lucknow, India; 2 Department of Pharmacology and Therapeutics, Chhatrapati Shahuji Maharaj Medical University, Chowk Lucknow, India

**Keywords:** Antiviral Agents, RNA Viruses, Hepatitis Delta Virus

## Abstract

**Background:**

The small delta antigen protein of hepatitis delta virus (HDV) has been shown to be important for replication of the virus and essential for the viral life cycle. Therefore, it may be an appropriate target for designing biological experiments for drug development to identify the potential inhibitors of hepatitis D.

**Objectives:**

To identify a novel molecule as possible drug candidate for the treatment of Hepatitis D.

**Materials and Methods:**

In the present study, a computational approach was used for the identification of novel small-molecule inhibitors against HDV replication using docking studies. An Autodock tool was used for docking and identifying the active binding sites in target proteins. The Lipinski filter and preADMET program were also used for determining the pharmacokinetic properties in order to filter out potential ligand molecules to restrain virus replication.

**Results:**

Our results suggest that pyridinone (3-[(4,7-dichloro-1,3-benzoxazol-2-yl) methylamino]-5-ethyl-6-methyl-pyridin-2(1H)-one) is a validated potential inhibitor of HDV replication and could be as a novel antiviral drug for the treatment of hepatitis D.

**Counclusions:**

We have identified a novel antiviral drug by using innovative computational approaches. The results provide a basis to experimentally develop into drug which can be used for the treatment of delta hepatitis.

## 1. Background

Delta hepatitis currently affects about 20 million people worldwide [1]. It is more prevalent among populations using injectable drugs, particularly, in countries bordering the Mediterranean Sea, while it is least common in Eastern Asia, although it is present in Taiwan, China, and India. Most child cases of delta hepatitis have been identified in Italy and Greece, and a few have been identified in northern Africa. The disease is caused by hepatitis delta virus (HDV), which was discovered in 1977 by Rizzetto and colleagues while they were studying liver biopsy samples of patients with hepatitis B surface antigen (HBsAg)-positive chronic liver disease [2]. HDV is an RNA virus and subviral satellite of hepatitis B virus, on which it is dependent for its envelope proteins [3]. The HDV genome, the smallest among animal pathogens, is a single-stranded negative sense circular RNA of about 1,700 nucleotides in length that forms a highly base-paired rod-like structure [4]. The HDV genome has a single open reading frame that encodes a single protein, the delta antigen protein (dAg). There are 2 forms of the delta antigen. The small form (195 amino acid long) is essential for HDV replication, and the large form, which has a 19-amino-acid extension at the carboxyl end (214 amino acid long), is crucial for virion packaging.

Assembly of HDV in infected human hepatocytes involves the association of the single-stranded genomic RNA with multiple copies of both small and large forms of the delta antigen protein to form a ribonucleoprotein particle, which in turn interacts with envelope proteins of the natural helper virus, hepatitis B virus, for the initiation of a new round of replication [5]. During HDV replication, 3 HDV RNA species are produced: the 1.7-kb antigenome, 1.7-kb genome, and 0.8-kb antigenomic-sense RNA. The former 2 RNA species form circular RNA and represent the replication products of the HDV RNA genome. The 0.8-kb RNA, however, is polyadenylated and thus resembles cellular pol II transcripts. This RNA acts as the mRNA for the translation of HDAg [6]. In HDV-infected cells, both small (S-HDAg) and large (L-HDAg) forms of HDAg are found [7][8][9][10]. Both forms are translated from the same open reading frame present on the 0.8-kb mRNA; the large form results from an RNA editing event [11][12][13], extending the S-HDAg open reading frame by 19 amino acids to encode the L-HDAg. The S-HDAg is required for HDV RNA replication in vivo [14]. In contrast, the L-HDAg inhibits the replication of HDV RNA [15][16]. The three-dimensional structure of S-HDAg protein of HDV was previously designed using threading by using a homology modeling approach. The model was evaluated according to the geometric design, fold recognition pattern, and compliance to the criteria for a quality model [17], which was used as a receptor in the present study.

Identification of new drug-like candidates is a crucial step in the early phase of drug discovery. The primary goal is to select a small number of compounds with desired properties (i.e. bioactivity against a drug target) from hypothetically available screening compounds [18]. The number of synthetically accessible organic molecules has been estimated to be in the range of 1,060 to 10,100 [19][20]. Hence, the comprehensive screening of such large number of compounds is evidently impossible. Advancement in high-throughput screening (HTS) and parallel synthesis since the early 1990s have accelerated the pace for developing of active molecules [21], and large compound libraries can be developed in a combinatorial fashion and screened with the help of robotics [22]. HTS is considerably costly and does not always yield many validated hits [23][24][25]. On the other hand, computational approaches like similarity searches [26], pharmacophore searching [27], molecular docking [28], quantitative structure-activity relationship (QSAR) methods [29], and de novo design [30] are useful to select tens to hundreds of compounds with predicted desired activity. Computational methods are successfully being applied in the selection and prioritization of putative drug target genes, computational modeling and X-ray structure validation of protein targets with drug lead compounds, simulated docking and virtual screening of potential lead compounds, and lead validation, etc., to develop new antiviral drugs. By facilitating the identification of active sites, characterization of conserved residues and, where relevant, prediction of catalytic residues, bioinformatics provides information that helps in designing selective and efficacious drug-like molecules [31].

## 2. Objectives

The aim of present study was to identify a novel antiviral molecule against HDV using virtual screening and docking strategies. An effective drug against hepatitis D has not yet been identified. Hence, there is a need to identify a suitable candidate for targeting HDV. We hope that our study results enable the identification of a new drug molecule.

## 3. Materials and Methods

### 3.1. Retrieval of the Three-Dimensional Structure of S-HDAg

The three-dimensional structure model of small delta antigen (S-HDAg) protein of HDV was retrieved from Protein Model Database (http://mi.caspur.it/PMDB/) as PM0075974 and used as a receptor for docking in the present study.

### 3.2. Ligand Selection

Several replication inhibitors were chosen from the National Centre for Biotechnology Information (NCBI) PubChem compound database as ligand molecules having the ability to inhibit the replication of S-HDAg protein of HDV. These molecules were downloaded in Structure-Data File (SDF) format and converted to Protein Data Bank (PDB) coordinates by using Open Babel (http://openbabel.org) converter. The selected ligand molecules were passed through the Lipinski filter (http://www.scfbio-iitd.res.in/utility/LipinskiFilters.jsp) for identifying their drug-like properties and only the molecules that passed through this filter were used for further analysis.

### 3.3. Receptor and Ligand Optimization

PDB coordinates of the small delta antigen protein and ligand molecules were optimized using Gromacs 4.0 suite [32] force field analysis and UCSF Chimera (http://www.cgl.ucsf.edu/chimera) tools, respectively. The optimized structures had minimum energy confirmation, which provided stability to the structure. These optimized receptor and ligand molecules were used for the docking study.

### 3.4. Docking Setup

Automated docking was used to determine appropriate binding orientations and conformations of various inhibitors at the target site. Autodock 4.0 [33] was used for docking of inhibitor molecules with S-HDAg protein of HDV, and Lamarckian Genetic Algorithm (LGA) was used to determine the globally optimized confirmation. Polar hydrogen atoms were added, and Kollman charge, atomic solvation parameters, and fragmental volumes were assigned to the protein using Autodock tools. The grid spacing was 0.375 Å for each spacing; each grid map consisted of 60 × 60 × 60 grid points, and 57.748, 57.623, and 57.694 coordinates. During each docking experiment, 25 runs were performed, and the population size was set at 150; maximum number of evaluation, 2,500,000; maximum number of generations, 27,000; rate of gene mutation, 0.02; and cross-over rate, 0.8. The remaining parameters were set as default. A root mean square deviation (RMSD) tolerance for each docking was set at 2.0 Å. Every inhibitor molecule had 0.274 coefficients of torsional degrees of freedom for docking. At the end of docking, a cluster analysis was performed. For docking of each ligand, all the confirmations were clustered together and ranked by the lowest binding energy. These docked complexes were subjected to further analysis. Autodock Vienna [34] and Patchdock tools [35] were used to check the accuracy of the results.

### 3.5. ADME/T Properties Calculation

Absorption, Distribution, Metabolism, Excretion, & Toxicity (ADME/T) properties of the selected inhibitor molecules were calculated using the preADMET online server (http://preadmet.bmdrc.org/) and PK/DB tool [36]. This program calculates the human intestinal absorption, in vitro Caco-2 cell permeability, Maden Darby Canine Kidney (MDCK) cell permeability, skin permeability, plasma protein binding, blood brain barrier penetration, and carcinogenicity.

## 4. Reults

Innovative computer-assisted approaches have been applied to identify new antiviral agents. The S-HDAg functions as a trans-activator of HDV replication cycle. The three-dimensional structure of S-HDAg protein of HDV was used in this study as a receptor. The length of the S-HDAg protein sequence is 195 amino acid; expected weight, 21,936.6 Da; and isoelectric point (pI), 10.02.

### 4.1. Screening and Optimization of Inhibitors

**Figure 1:**
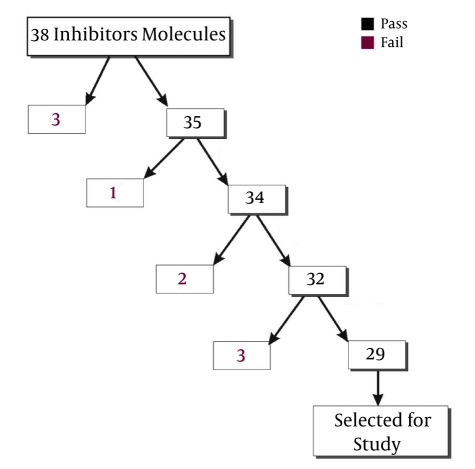
Flow Chart of Screening for Inhibitors.

The S-HDAg protein of HDV has been reported to play a major role in the replication process. Therefore, replication inhibitors were required to block the replication process. We selected 38 replication inhibitors from the PubChem compound database as ligand molecules. Details of the selected molecules and their physiochemical properties, drug-like properties, and 2D structures are given in supplementary Table 1. Some inhibitors that did not follow the 5 Lipinski rules, -i.e., not more than 5 hydrogen bond donors, not more than 10 hydrogen bond acceptors, molecular weight not greater than 500 daltons, and an octanol-water partition coefficient log P of not more than 5 [37], or those that had a polar surface area of less than 140 Ǻ, as suggested by Arup et al. [38], were discarded at various steps as shown in the flow chart in Figure 1. After this filtration step, only 29 molecular inhibitors remained that were used for further analysis.The PDB coordinates of the S-HDAg protein (as receptor) and inhibitor (as ligand) molecules were optimized using Gromacs and Chimera tools to attain their minimum energy confirmation and obtain a thermodynamically stable structure. Next, the receptor and ligands were subjected to docking using Autodock 4.1.

**Table 1:** Autodock Binding Free Energies Calculated Using Different Energy Solution and Inhibition Constants

**SN**	**PubChem_Id**	**∆Gtors a**	**∆Gvdw a+ ∆Ghbond^a^ + ∆Gdesolv a**	**∆Gelec a**	**∆Gintermol a**	**∆Gbinding a**	**∆Ginter a**	**Ki a**	**RMSD a**
1.	CID_546	1.49	-3.00	-3.23	-6.23	-5.35	-1911.90	120.66	101.968
2.	CID_3043	0.60	-5.29	-0.32	-5.61	-5.52	-1912.09	89.46	102.720
3.	CID_3414	0.30	-0.91	-5.30	-6.20	-5.91	-1912.93	46.81	96.234
4.	CID_3415	1.19	-2.96	-2.87	-5.83	-5.32	-1912.32	126.25	103.468
5.	CID_3963	1.49	-6.63	-0.09	-6.72	-6.74	-1912.54	11.47	105.272
6.	CID_5718	0.89	-5.59	-0.31	-5.90	-5.78	-1912.77	57.55	98.761
7.	CID_5726	1.19	-5.69	-0.16	-5.85	-5.66	-1912.22	71.04	105.693
8.	CID_24066	0.60	-5.11	-0.14	-5.25	-5.47	-1912.31	98.30	101.706
9.	CID_35370	1.19	-6.89	-0.24	-7.13	-6.95	-1912.61	7.99	104.924
10.	CID_47318	0.00	-5.37	0.03	-5.34	-5.34	-1912.42	121.58	105.406
11.	CID_50599	0.60	-5.45	-0.36	-5.81	-5.52	-1912.19	89.79	102.718
12.	CID_60172	2.39	-5.50	-1.43	-6.94	-5.37	-1911.75	114.91	101.710
13.	CID_64993	2.09	-6.76	-0.17	-6.92	-6.15	-1912.26	30.91	105.601
14.	CID_65002	1.19	-8.31	0.14	-8.17	-7.55	-1913.21	2.92	99.881
15.	CID_449080	1.49	-6.98	0.03	-6.94	-6.69	-1912.75	12.39	105.045
16.	CID_451515	1.19	-5.72	-0.14	-5.86	-5.91	-1911.91	46.93	107.086
17.	CID_455007	1.19	-6.97	-0.16	-7.12	-6.94	-1912.51	8.16	105.024
18.	CID_455194	1.49	-6.82	-0.06	-6.88	-6.73	-1912.59	11.58	105.853
19.	CID_455271	0.60	-5.15	-0.19	-5.34	-5.53	-1912.26	88.89	106.698
20.	CID_455661	0.89	-5.77	-0.13	-5.90	-5.68	-1912.25	68.08	107.475
21.	CID_456314	0.60	-5.66	-0.27	-5.93	-6.19	-1913.20	28.86	106.348
22.	CID_676643	0.60	-5.63	-0.32	-5.95	-6.20	-1913.02	28.77	98.684
23.	CID_3246700	1.19	-7.02	-0.33	-7.34	-6.38	-1912.57	21.21	103.764
24.	CID_4451056	0.60	-1.39	-6.14	-7.53	-5.31	-1911.55	127.36	94.365
25.	CID_5742630	0.60	-5.24	-0.25	-5.48	-5.69	-1912.30	67.92	107.627
26.	CID_10198219	0.89	-5.59	-0.30	-5.90	-5.78	-1912.61	57.55	98.729
27.	CID_11778134	2.39	-5.92	-1.31	-7.23	-5.75	-1911.95	61.19	101.217
28.	CID_16219192	0.60	-5.44	-0.39	-5.83	-5.44	-1912.14	103.45	102.652
29.	CID_169159	0.60	-6.29	-0.23	-6.52	-5.90	-1912.33	47.44	104.725

^a^ Abbreviations: ΔGbinding, estimated binding free energy (kcal/mol); ΔGdesolv, desolvation factor of binding free energy (kcal/mol); ΔGelec, electrostatic factor of binding free energy (kcal/mol); ΔGhbond, H-bonding factor of binding free energy (kcal/mol); ΔGinter, Gibbs free energy of binding (kcal/mol); ΔGintermol , intermolecular energy (kcal/mol); ΔGtors, torsional energy of binding (kcal/mol); ΔGvdw, Vander wall or Lennard-Jones potential factor of binding free energy (kcal/mol); Ki, inhibition constant (μM); RMSD, reference root mean square deviation

### 4.2. Docking and Active Site Studies

Autodock 4.1 was used to dock inhibitors to identify the active entities and determine the active binding sites in target proteins. Lamarckian Genetic Algorithm (LGA) for docking was implemented with defined parameters for determining the docking performance. The output of molecular docking was clustered to determine the binding free energy (BE) and optimal docking energy conformation that is considered as the best docked structure, as well as to elucidate their binding state in the receptor. BE for each docking was calculated using a semi-empirical free energy force field with charge-based desolvation and grid-based docking. The force field was decided on the basis of a comprehensive thermodynamic model that allows the incorporation of intermolecular energies into the predicted BE [33]. It also included a charge-based method for the evaluation of desolvation. The method was designed to use a typical set of atom types. The formula for calculating semiempirical BE is given below:

∆Gbinding = ∆Gvdw + ∆Gelec + ∆Ghbond+ ∆Gdesolv + ∆Gtors + ∆Gintermol, where ∆Gvdw = Vander wall or Lennard-Jones potential, ∆Gelec = electrostatic factor with distance-dependent dielectric, ∆Ghbond = H-bonding potential with directionality, ∆Gdesolv = charge-dependent variant of volume-based atomic solvation, ∆Gtors = torsional energy based on the number of rotatable bonds, and ∆Gintermol = intermolecular energy of protein and ligand molecules [33]. The summations were performed over all pairs of ligand and protein atoms, and the BE was calculated. Docking was also performed to determine the inhibition constant (Ki) for drug-like molecules and to calculate the RMSD value.

**Figure 2:**
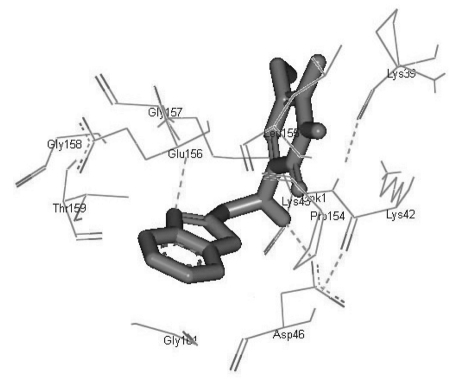
H-Bonds in Inhibitor CID_65002

**Figure 3:**
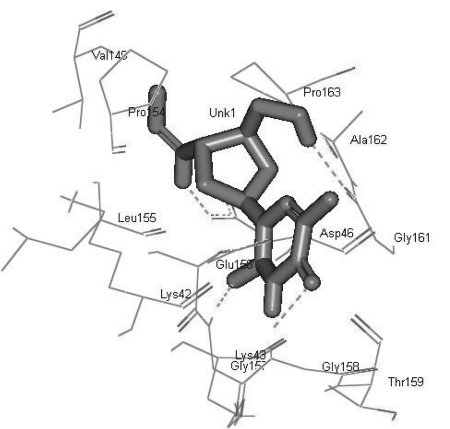
H-bonds in Inhibitor CID_35370

**Figure 4:**
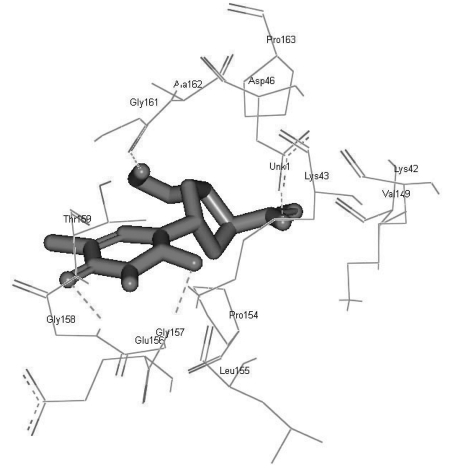
H-Bonds in Inhibitor CID_455007

Most docked inhibitors interacted in the same fashion and showed more hydrogen bonding with GLU156, GLY157, GLY158, GLY161, and ASP46 amino acids (Figures 2, 3, and 4). The binding modes and geometrical orientation of all compounds were almost identical, suggesting that all the inhibitors occupied a common cavity in the receptor. The binding pattern of each inhibitor molecule with an active site and the hydrogen bond distance in the target protein are shown in supplementary Table 2. Autodock BEs (∆Gbind, kcal/mol), calculated using different energy solution and inhibition constants for each protein-ligand complex are shown in Table 1. Among the molecules tested, pyridinone (CID_65002) showed the lowest BE, i.e., -7.55 kcal/mol. In other words, it had the highest potential binding affinity for the binding site of the target protein.

**Table 2:** Comparative Docking Result Obtained with Various Docking Tools (Energy is Represented in Kcal/mol)

**SN**	**PubChem_Id**	**Autodock 4.0**	**Autodock Vienna**	**Patchdock**
1.	CID_65002	-7.55	-8.0	-44.35
2.	CID_35370	-6.95	-7.2	-34.82
3.	CID_455007	-6.94	-7.2	-35.10

For confirming the accuracy of the predicted result, the Autodock Vienna and Patchdock tools were also used for conducting docking studies using the parameters mentioned above. Autodock Vienna generates a genetic algorithm and calculates the binding affinity for the binding site of a target protein. On the other hand, the Patchdock tool is a geometry-based molecular docking algorithm that identifies docking transformations that yield good molecular shape complementarities, can also perform clustering, and calculates the global BE. The clustering RMSD value was considered as 2.0 for this analysis. Pyridinone showed good binding affinity, i. e. -8.0 Kcal/mol with the protein and a minimum global free energy of -44.35 Kcal/mol, as revealed by Autodock Vienna and Patchdock tool, respectively Table 2. Hence, in the present study, pyridinone was confirmed to be an appropriate molecule by using 3 docking tools, and it might be considered as an antiviral drug candidate in future studies.

### 4.3. Verification of Inhibitors as Suitable Drugs

The selected ligand molecules were then subjected to analysis for their pharmacokinetic properties. ADME/T was calculated using the preADMET tool, which also contains various parameters to identify a potential drug candidate. This tool identified pyridinone out of the 29 selected ligands as a potential drug candidate. Toxicity analysis of pyridinone yielded negative carcinogenicity results in both mouse and rat models. ADME/T calculation revealed that pyridinone had human intestinal absorption (%) of 94.65; in vitro Caco-2 cell permeability (nm/s), 22.47; in vitro MDCK cell permeability (nm/s), 7.10; in vitro skin permeability (logKp·cm/h), -4.55; in vitro plasma protein binding (%), 61.24; and in vivo blood brain barrier penetration (c.blood/c.brain), 0.13. ADME/T properties of the selected molecule were also predicted using the PK/DB database. The results of this analysis validated all ADME/T parameters for the ligand, confirming it to be considered a drug candidate. Pyridinone has already been identified as an antiviral agent and is also a good replication inhibitor. Thus, it fulfills the criteria to be considered as a possible drug candidate for the treatment of delta hepatitis.

## 5. Discussion

Hepatitis D is associated with mortality and morbidity, worldwide and therefore, many treatment strategies for hepatitis D have been accepted, but none of them have been found to be effective. Successful utilization of computational tools and resources has benefited the drug discovery studies. Computational approaches have yielded noteworthy and reproducible results with regard to drug discovery; and several drugs have been identified using these approaches. These includes flavivirus inhibitors [39], antimalarial agents [40], anti-influenza molecules [41], antiSARS drug [42], antiHIV drug [43], Our computational analysis suggests that pyridinone can be considered as a potential candidate drug for hepatitis D. The present study aimed to identify a novel inhibitor against HDV by using structure-based drug designing approach. A docking study conducted for identifying target proteins using known computational tools provided a clue regarding the molecules that interacted with possible inhibitor molecules that inhibited the virus replication and thereby could be used for treating delta hepatitis. These results would be beneficial to all researchers and pharmaceutical individuals who are conducting studies to identify treatment strategies for delta hepatitis.
